# The Novel Role of PGC1α in Bone Metabolism

**DOI:** 10.3390/ijms22094670

**Published:** 2021-04-28

**Authors:** Cinzia Buccoliero, Manuela Dicarlo, Patrizia Pignataro, Francesco Gaccione, Silvia Colucci, Graziana Colaianni, Maria Grano

**Affiliations:** 1 Department of Emergency and Organ Transplantation, University of Bari, 70124 Bari, Italy; cinzia.buccoliero@uniba.it (C.B.); patrizia.pignataro@uniba.it (P.P.); fra.gaccione@gmail.com (F.G.); graziana.colaianni@uniba.it (G.C.); 2 Department of Basic Medical Sciences, Neuroscience and Sense Organs, University of Bari, 70124 Bari, Italy; manuela.dicarlo@uniba.it (M.D.); silviaconcetta.colucci@uniba.it (S.C.)

**Keywords:** mitochondria, bone metabolism, metabolic regulations

## Abstract

Peroxisome proliferator-activated receptor gamma coactivator 1-alpha (PGC1α) is a protein that promotes transcription of numerous genes, particularly those responsible for the regulation of mitochondrial biogenesis. Evidence for a key role of PGC1α in bone metabolism is very recent. *In vivo* studies showed that PGC1α deletion negatively affects cortical thickness, trabecular organization and resistance to flexion, resulting in increased risk of fracture. Furthermore, in a mouse model of bone disease, PGC1α activation stimulates osteoblastic gene expression and inhibits atrogene transcription. PGC1α overexpression positively affects the activity of Sirtuin 3, a mitochondrial nicotinammide adenina dinucleotide (NAD)-dependent deacetylase, on osteoblastic differentiation. *In vitro*, PGC1α overexpression prevents the reduction of mitochondrial density, membrane potential and alkaline phosphatase activity caused by Sirtuin 3 knockdown in osteoblasts. Moreover, PGC1α influences the commitment of skeletal stem cells towards an osteogenic lineage, while negatively affects marrow adipose tissue accumulation. In this review, we will focus on recent findings about PGC1α action on bone metabolism, *in vivo* and *in vitro*, and in pathologies that cause bone loss, such as osteoporosis and type 2 diabetes.

## 1. Introduction

The peroxisome proliferator-activated receptor γ (PPARγ) and coactivator-1s (PGC-1s) are members of a family of transcriptional coactivators consisting of PGC1α, Peroxisome proliferator-activated receptor gamma coactivator 1-beta (PGC1β), and PGC-1 related coactivator (PRC), all of which play key roles in the regulation of mitochondrial biogenesis in all tissues [1]. PGC1α, upon activation through phosphorylation or deacetylation, coordinates the regulation of the nuclear respiratory factors 1 and 2 (Nrf1 and Nrf2) activity, along with mitochondrial transcription factor A (Tfam) expression [2].

Genome-wide association studies (GWAS) in humans and studies in mice carrying mitochondrial DNA (mtDNA) mutations have suggested that defective mitochondria or damaged mtDNA are associated with osteoporosis [3,4]. Along these lines, mitochondrial dysfunction increasingly appears to be one of the most important cellular features driving the aging process [5]. Mitochondria play a key role in the differentiation of bone precursor cells. During osteogenesis, osteoblasts significantly increase their mitochondrial biogenesis, the activity of complex I in the mitochondrial electron transport chain and the content of adenosine triphosphate (ATP) [6]. Bone marrow macrophages and mature osteoclasts also show increased mitochondrial protein content [7].

Although the role of PGC1α in high-energy expenditure tissues has been well documented, its activity on bone tissue has long remained unknown. However, considering the importance of the mitochondrial biogenesis process also in bone metabolism, great efforts have been made in recent years to decipher the effects of PGC1α activity in bone cells. The first study, demonstrating that PGC1α, stimulated by parathyroid hormone (PTH), synergizes with nuclear related receptor-1 (Nurr1) to transactivate target genes in osteoblastic cells, dates back to 2006 [8]. Several studies both *in vitro* and *in vivo* on mouse models with PGC1α deletion in the whole-body or with conditional deletion in skeletal stem cells or osteoblasts have since been performed (Table 1).

In this review, we aim to summarize the current knowledge of the role of PGC1α as an anabolic factor in bone metabolism in both physiological condition and bone related pathologies, with the focus on paving the way for further studies in the future.

## 2. The Bone Phenotype of PGC1α Knock-Out Mice

**Figure 1:**
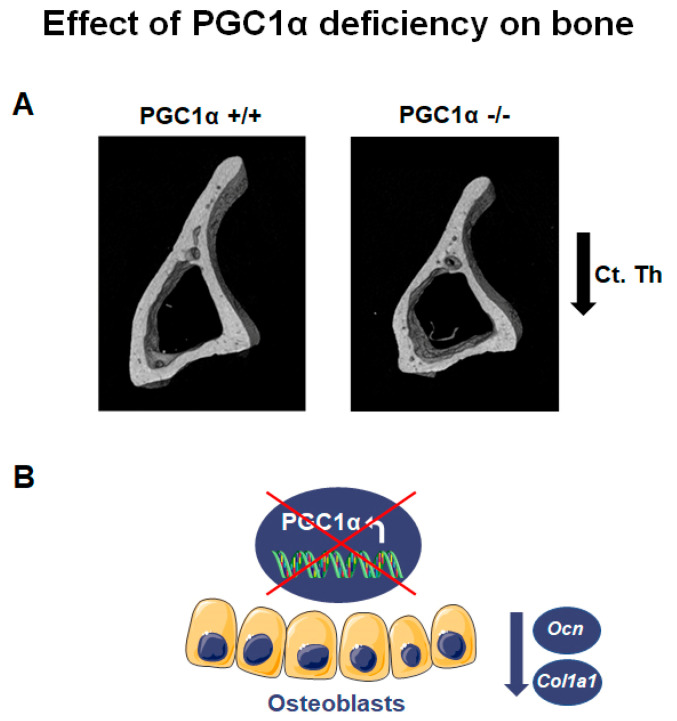
PGC1α deletion affects bone. (**A**) Representative images of micro-CT-generated sections of the tibia midshaft of PGC1α+/+ and PGC1α−/− mice show a reduction in cortical thickness (Ct. Th) in the absence of PGC1α. Adapted from [12]. (**B**) Schematic representation of the effect of PGC1α deletion in osteoblasts consisting of the reduction of *Ocn* and *Col1a1* levels.

Global deletion of PGC1α has a pronounced impact on bone phenotype, particularly in adulthood. PGC1α deficient mice aged 12 months showed lower bone mass and strength than wild-type littermates [12]. PGC1α loss compromised long bones, especially the tibia, causing a reduction in cortical bone mass and strength [12]. While trabecular thickness (Tb. Th) decreased in PGC1α knock-out mice, trabecular number (Tb. N) increased compared to wild-type mice, thus enhancing anisotropy degree [12]. The anisotropy, notably in bone tissue, is a parameter also used in humans to detect the degree of material organization, thus defining the relationship between architectural structure and mechanical properties of bone [15]. Increased degree of anisotropy was observed in postmenopausal women with vertebral fracture compared with age-matched control cases, suggesting that fracture risk assessment can be improved after acquiring information related to the organization of trabecular bone architecture [16]. Moreover, PGC1α deficiency resulted in modification of the trabecular pattern and reduction of cortical thickness (Ct. Th) (Figure 1A). Furthermore, the deficiency also reduced resistance to flexion (~48.4%), implying PGC1α importance in preventing the risk of fracture [12]. Furthermore, the absence of PGC1α *in vivo* caused a reduction of the bone-matrix protein Osteocalcin (Ocn) (Figure 1B) [12], in accordance with a previous *in vitro* study, which showed that PGC1α contributed to the activity of osteoblasts, inducing together with Nurr1 the expression of Ocn [8]. Notably, Ocn promoter contains three Estrogen-related receptor alpha (ERRα) response elements, and ERRα was thought to cooperate with PGC1α to regulate gene expression involved in mitochondrial pathways and oxidative phosphorylation [17]. In addition, ERRα interacts with PGC1α to ameliorate the Ocn promoter functionality [9].

Moreover, bone marrow precursors of PGC1α deficient mice expressed a lower mRNA level of collagen type I α 1 (*Col1a1*), the most abundant bone matrix protein [18], than wild-type mice (Figure 1B) [12]. Consistently, bone marrow cells from PGC1α knock-out mice cultured *ex vivo* displayed a delayed differentiation of osteoblasts [12]. Interestingly, osteoclasts from PGC1α deficient mice, differentiated from pure monocyte cultures, also showed delay in the differentiation process [12]. In contrast, when osteoclasts from PGC1α null mice were differentiated from a culture of whole bone marrow, an increased formation of multinucleated osteoclasts was observed [12]. This result suggested that the elevated Receptor activator of nuclear factor kappa-Β ligand (RANKL) levels observed in bone marrow of knock-out mice could be the indirect mechanism through which osteoblasts increase osteoclast formation and activity *in vivo*.

Moreover, in agreement with Lin and colleagues’ study of 2004 [19], PGC1α knock-out mice had 30% lower weight than control mice, lower ratio of inguinal white adipose tissue (iWAT)/body weight and strong decrease (~75%) in adipocyte area [12]. Colaianni and colleagues also evaluated, in iWAT, the uncoupling protein 1 (Ucp1) expression, considering its importance as a master gene involved in the trans-differentiation program from white adipocytes to adipocytes with a brown adipose tissue (BAT)-like phenotype [20]. PGC1α deficiency negatively affected *Ucp1* expression also in iWAT and not only in the interscapular brown fat, as previously shown [12,21].

Although the overall results of this study highlight for the first time that PGC1α plays a critical role in the regulation of bone mass, one limitation may be that the characterization of the bone phenotype may have been masked by other secondary systemic effects due to whole-body PGC1α deletion. Similarly, it is plausible that the 30% reduction in body weight in PGC1α knock-out mice affected the mechanical loading on their skeleton. Therefore, the generation of conditional PGC1α knock-out models, with specific deletion in osteoblasts or osteoclasts, will be required to provide further understanding of the contribution of this transcription factor to bone metabolism.

## 3. PGC1α/β Role in Modulating Osteoblast and Osteocyte Gene Expression

Ding and colleagues, in 2017, published a study on the effect of PGC1α overexpression on Sirtuin 3 (SIRT3) knockdown in murine osteoblast cell line (MC3T3-E1) [10]. The Sirtuins (SIRTs), which are characterized by a sirtuin core domain, are the family of NAD+-dependent deacetylase proteins that regulate numerous cellular processes including proliferation, apoptosis, autophagy and DNA repair [22]. Among the members of this family of proteins, SIRT3-5, expressed in mitochondria, influence the metabolic activity of these organelles. In particular, SIRT3 acts by deacetylating many proteins and regulating mitochondrial biogenesis and reactive oxygen species homeostasis. Of note, SIRT3 is involved in the control of ATP production in mitochondria by acting on the respiratory chain, suggesting a key role of SIRT3 as a crucial mediator for cellular energy production [22].

SIRT3 exhibits deacetylase activity and affects the regulation of many proteins with a key role in osteoblastic differentiation, maintaining bone homeostasis [22]. SIRT3 knockdown negatively affected alkaline phosphatase (ALP) activity and expression of the major gene involved in osteoblastic differentiation, Runt-related transcription factor 2 (*Runx2*), *Col1α1* and *Ocn* [10]. Moreover, in differentiated MC3T3-E1, SIRT3 knockdown inhibited mitochondrial function, evaluated by Complex I, II, III, IV, and V activity measurements, oxygen consumption and mitochondrial membrane potential level [10]. In addition, the expression of two key factors of mitochondrial biogenesis, *Nrf1* and *Tfam*, was negatively affected by the absence of SIRT3 [10]. Of note, mitochondrial size increased, and mitochondrial density decreased by SIRT3 deletion [10]. This study also demonstrated that SIRT3 knockdown reduced the expression, at both mRNA and protein levels, of superoxide dismutase 2 (SOD2), an efficiently mitochondrial molecule with antioxidant activity that converts superoxide to the less reactive hydrogen peroxide (H2O2) [10,23]. Overexpression of SOD2 markedly reverted reduction of oxygen consumption, ALP staining and *Runx2*, *Col1α1*, and *Ocn* mRNA level [10]. These findings indicated a key role of SOD2 in SIRT3 knockdown-induced inhibition of osteogenic differentiation and mitochondrial activity [10].

PGC1α overexpression restored the reduction of mitochondrial density, mitochondrial membrane potential, *Nrf1* and *Tfam* mRNA expression and ALP activity [10]. Moreover, PGC1α overexpression inverted the increase of mitochondrial size, highlighting a key role of PGC1α in SIRT3 activity on osteoblastic differentiation [10]. These findings were relevant to the most recent evidence confirming that the SIRT3-PGC1α-SOD2 interaction is the central pathway used by SIRT3 to regulate bone homeostasis [24].

Unlike SIRT3, SIRT4, and SIRT5, which are localized in the mitochondria, SIRT1, SIRT6, SIRT7 are localized predominantly in the nucleus. Specifically, SIRT1 deacetylates histones H3, H4, and H1, and modifies nonhistone proteins, such as the transcription factors p53, nuclear factor-κB (NF-κB), and the members of the class O of forkhead box transcription factors (FoxOs) [25]. The effects of SIRT1 on the skeleton have been extensively studied, and results obtained in mouse models have shown that SIRT1 increases trabecular bone mass by stimulating Wnt signaling in osteoblasts and osteocytes. During differentiation of these bone cells, SIRT1 deacetylates FoxOs by preventing FoxO association with β-catenin and potentiates Wnt signaling [25].

In a recent study, the role of PGC1α/β and its activators 5’ adenosine monophosphate-activated protein kinase (AMPK) and SIRT1 in osteocyte differentiation and reprogramming was investigated [11]. Preosteocytic cells (IDG-SW3), differentiated for 14 days in the presence of glucose, and femur-derived bone organotypic cultures, maintained in glucose media, were treated with 5-Aminoimidazole-4-carboxamide ribonucleotide (AICAR) and SRT2104, two chemical factors activating AMPK and SIRT1 pathway, respectively [11]. AMPK activation via AICAR treatment upregulated *Runx2* and *Osterix* in IDG-SW3 cells and the osteocyte genes Dentin matrix acidic phosphoprotein 1 (*Dmp1*), Fibroblast growth factor 23 (*Fgf23*), and Sclerostin (*Sost*) in both IDG-SW3 cells and bone organotypic cultures [11]. In parallel, treatment with SRT2104 activating SIRT1 stimulated the expression of late osteocyte markers. All together, these results suggested that activation of AMPK/SIRT1 plays a key role in osteocyte differentiation [11].

To evaluate PGC1α/β role in modulating osteoblast and osteocyte gene expression, retroviral pMSCV-PGC1α was used for PGC1α overexpression in primary osteoblasts and IDG-SW3 cells [11]. PGC1α/β deletion was performed using retroviral pMSCV-puro-Cre-ERT2, pMSCV-puro and pMSCV-GFP virus in primary osteoblasts and primary osteocytes derived from control mice [11]. Real-time quantitative polymerase chain reaction (qRT-PCR) analysis showed that PGC1α overexpression upregulated many key factors involved in osteoblast and osteocyte differentiation both in IDG-SW3 cells and primary osteoblasts, while PGC1α/β deletion strongly caused their reduction [11]. Moreover, micro-computed tomography analysis (μCT) of femurs from 8 week-old mice with specific deletion of Ppargc1α/β in osteoblasts (Ppargc1α/β^f/f^;Col1a1-Cre) showed a reduction of both cortical and trabecular parameters compared to control mice [11]. PGC1α/β deletion in osteoblasts decreased cortical bone volume (BV), bone area (B.Ar) and Ct. Th, while bone perimeter (B.Pm) was not affected [11]. In addition, BV/total volume (TV), Tb. N and Tb. Th were lower in the absence of PGC1α/β, while trabecular space increased [11].

Although a limitation of this study was that Pgc1α/β was deleted in both osteoblasts and osteocytes, and therefore the relative contribution of each transcription factors in the two bone cell types could not be deciphered, the overall results suggested a central role of PGC1s in bone metabolism and osteoblast and osteocyte differentiation.

## 4. PGC-1α Regulates Skeletal Stem Cell Fate and Its Deletion Increases Marrow Adipose Tissue (MAT) Accumulation

**Figure 2:**
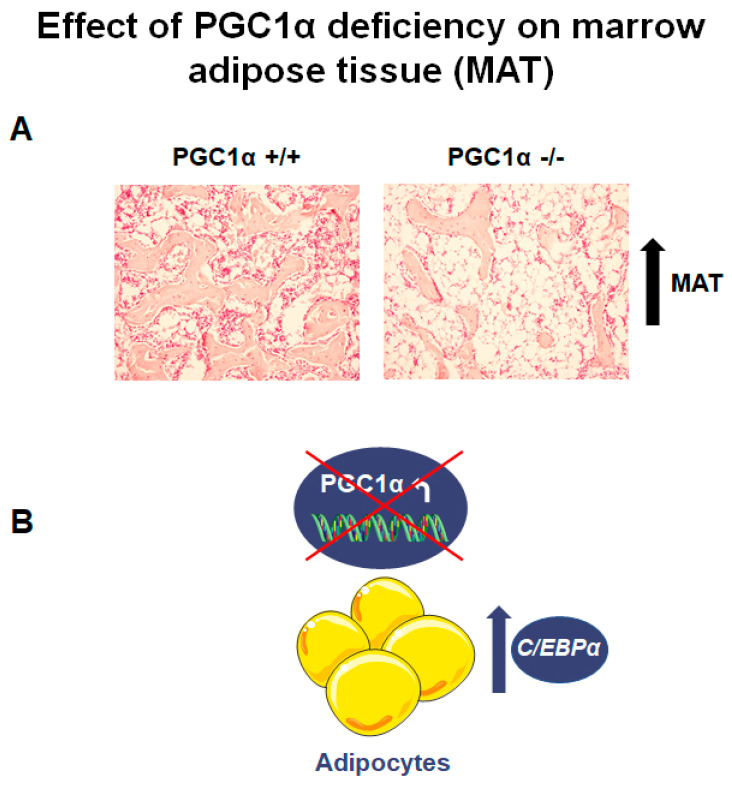
PGC1α deletion affects marrow adipose tissue (MAT). (**A**) Photomicrographs of hematoxylin and eosin-stained sections of MAT from PGC1α +/+ and PGC1α −/− (magnification: 20×) show an increased number of adipocytes in the absence of PGC1α *(unpublished data)*. (**B**) Schematic representation of the effect of PGC1α deletion in bone marrow adipocytes consisting of the increase of *C/EBPα* expression.

Yu and colleagues, in 2018, investigated PGC1α role in the commitment of skeletal stem cells (SSCs) and thus its involvement in the balance between bone tissue and adipose tissue associated with osteoporosis [13]. SSCs are bone marrow stromal cells characterized by the ability to self-renew and to differentiate, if properly stimulated, towards both the osteoblastic and adipocytic cell lineage [26,27,28]. In addition, several pieces of evidence, in both animal and human data, demonstrated an inverse association between MAT and bone density and strength [29]. In humans, MAT was negatively associated with bone mineral density (BMD) in healthy Caucasian women [30] and in a group of healthy Caucasian and African-American men and women aged 38–52 [31]. In animals, in two mouse models of type 1 diabetes mellitus, the adipocyte markers, Peroxisome proliferator-activated receptor γ2 (PPARγ2), adipocyte Protein 2 (aP2) and Resistin (RETN) were upregulated in tibia, while Ocn mRNA and osteocalcin serum level decreased [32,33]. Yu and colleagues’ study showed that PGC1α deficiency increased bone loss and MAT accumulation, in skeletal tissue during aging [13], supported by findings showing the increase of CCAAT/enhancer-binding protein alpha (C/EBPα), a key transcription factor involved in adipocyte differentiation [34], by bone marrow precursors from PGC1α deficient mice (Figure 2A,B) [12].

Pgc1α levels decreased in older subjects compared to those younger, both in bone marrow SSCs of 3- and 18-month-old mice and in human skeletal stem cells (hSSCs) of two age groups (20–40 and 70–90 years) [13]. Immunohistochemical staining and integral optical density analysis confirmed the lower expression of PGC1α in femurs of aged mice compared to younger mice [13]. Femurs of 18-month-old wild-type mice, analyzed by μCT, displayed a loss of ~40% of BMD and 50% of BV/TV compared to 3-month-old wild-type mice [13]. This bone loss was exacerbated in 18-month-old PGC1α knock-out mice, which showed higher reduction of BMD (~58%) and BV/TV (~60%) compared to the younger mice [13].

To understand the mechanisms underlying this bone loss, the authors showed that PGC1α deletion caused a downregulation in osteoblast number, osteoblast surface, circulating osteocalcin (67%), bone formation rate (BFR) and mineral apposition rate (MAR), while the number of osteoclasts increased [13]. On the other hand, PGC1α deficiency, as well as the process of aging, induced MAT accumulation, with a significant increase in adipocyte number and adipocyte area [13]. In addition, Yu et al. conditionally deleted PGC1α in SSCs using Prx1-Cre (Prx1;Pgc1α^f/f^) and evaluated whether this deletion influenced osteoporotic bone loss and MAT accumulation in mice after ovariectomy, which mimics post-menopausal osteoporosis [13]. In ovariectomized mice with specific deletion of PGC1α in skeletal stem cells (Prx1;Pgc1a^f/f^ mice), BMD (60%) and BV/TV (54%) were significantly decreased compared to sham mice [13]. Bone mass was impaired in the absence of PGC1α after ovariectomy due to an exacerbated defect in bone formation as demonstrated by reduced serum levels of osteocalcin as well as rates of bone formation and mineral apposition [13]. At the same time, PGC1α deletion associated with ovariectomy promoted estrogen deficiency-induced MAT accumulation [13]. Moreover, by decreasing the expression of the pro-inflammatory cytokine, the interleukin-6 (Il-6), PGC1α also controls osteoclast activity and bone resorption through inhibition of NF-κB activation [13].

The effects of PGC1α deletion on osteoblast and adipocyte differentiation were also confirmed in *in vitro* study using SSCs derived from PGC1α knock-out mice [13]. These cells, cultured *in vitro*, showed reduced ALP activity and mineralized nodule formation, while adipogenesis was enhanced [13]. Interestingly, PGC1α deletion in SSCs significantly decreased the expression of the transcriptional coactivator with a PDZ-binding domain (TAZ), during osteogenic differentiation [13]. TAZ modulates hSSCs commitment toward an osteogenic lineage, coactivating osteoblastic gene expression with Runx2, while inhibiting PPARγ-related gene transcription [35,36,37]. However, PGC1α deletion did not affect the induction of yes-associated protein 1 (YAP1), another co-effector with TAZ of the tumor suppressor Hippo pathway [13].

Although the role of PGC1α in osteoclastogenesis remains unclear, data indicate that deletion of PGC1α in SSCs could indirectly promote osteoclastogenesis and bone resorption by increasing the expression of pro-inflammatory cytokines. The existence of a connection between PGC1α loss and inflammation-induced dysregulation of SSC fate suggests that induction of PGC1α would be a promising potential therapeutic approach for the prevention of osteoporosis. It is desirable that future studies will investigate mouse models with conditional deletion of PGC1α in osteoclast precursors to determine whether protection from ovariectomy-induced bone loss following PGC1α induction depends on suppression of osteoclastic bone resorption.

## 5. PGC1α Role in Bone-Related Pathologies

Since PGC1α expression in skeletal muscle is suppressed in diabetes [38,39], its role has been also evaluated in type 2 diabetes, a pathological condition that increases bone loss and fracture risk [14,40,41]. Expectedly, μCT analysis in femur epiphyses of leptin receptor-deficient diabetic mice showed an age- and genotype-dependent compromised trabecular network compared to control mice [14]. BMD, BV/TV, Tb. N, Tb. Th, and connectivity density (Conn.D) decreased, while trabecular separation (Tb. Sp) increased [14]. In addition, all cortical parameters measured in diabetic mice were negatively affected compared to control group [14]. Interestingly, leptin receptor-deficient diabetic mice displayed lower levels of Ocn, Adiponectin (AdipoQ), Runx2 and PGC1α, than wild-type mice [14]. Both diabetes and obesity increase atrogenes involved in protein catabolism, which negatively affects muscle health [42], whereas PGC1α activation downregulates atrogene expression and prevents muscle atrophy under different stresses [43]. Indeed, Muscle RING-finger protein-1 (*Murf1*), cathepsin L (*Ctsl*) and *Atrogin-1* were upregulated in leptin receptor-deficient diabetic mice during aging compared with control mice [14]. AdipoQ, an anti-diabetic adipokine, and adiponectin receptor 1 (AdipoR1) modulate PGC1α expression [44]. Khan and colleagues treated leptin receptor-deficient diabetic mice with an AdipoR1 agonist, 6-*C*-β-d-glucopyranosyl-(2S,3S)-(+)-5,7,3′,4′-tetrahydroxydihydroflavonol (GTDF), for 4 weeks [14]. They observed an increase in femurs of BV/TV, Tb. N, Tb. Th and a decrease in Conn.D and Tb. Sp compared to untreated mice [14]. In addition, femurs of leptin receptor-deficient diabetic mice treated with GTDF displayed a strong decrease in Murf1 and increase in resistance to bending. The increased periosteal bone lining cell number, along with *Runx2* and *Ocn* upregulation was accompanied by an increase of PGC1α level and phosphorylated AMPK (pAMPK), a key mediator of AdipoR1 pathway, which in turn activates PGC1α [14]. By silencing PGC1α in mouse calvarial osteoblasts, the GTDF-mediated induction of ALP activity was drastically inhibited [14]. These findings suggested that bone loss in leptin receptor-deficient diabetic mice could be suppressed by GTDF through the activation of PGC1α, which stimulates osteoblastic gene expression and inhibits transcription of atrogenes [14].

Of note, this study demonstrated that, as in skeletal muscle, a reciprocal relationship between Murf1 and PGC1α also exists in osteoblasts. Future studies should investigate the possible interaction of Murf1 with the transcription factors Runx2 and PGC1α to understand whether the reduction in osteoblast function in leptin receptor-deficient diabetic mice occurs because of suppression of PGC1α and induction of atrogenes.

## 6. Conclusions

Although several studies have investigated the role of PGC1α in various cellular processes, especially in tissues with high energy expenditure, its action on bone metabolism has not yet been fully investigated. However, great efforts have been made in the last decade to decipher the effects of PGC1α on bone, both *in vitro* and *in vivo*. *In vitro*, PGC1α positively stimulates SIRT3 activity on osteogenic differentiation [10] and increases the expression of osteocyte master genes [11]. Moreover, PGC1α regulates SSC fate and its deletion increases MAT accumulation [13]. Furthermore, PGC1α absence in ovariectomized mice caused a reduction in BFR and MAR, as well as in Ocn serum levels, thus indicating a severe defect in bone formation [13]. *In vivo*, in aged mice, PGC1α deficiency negatively regulates bone mass and strength [12], while in diabetic mouse model, PGC1α activation reverses osteopenia resulting from diabetic phenotype [14].

However, many aspects still need to be investigated regarding the PGC1α role as an anabolic factor in bone metabolism, in both physiological conditions and bone-related pathologies.

## Figures and Tables

**Table 1:** PGC1α activity on bone metabolism *in vitro* and *in vivo*.

PGC1α Activity on Bone Metabolism	
*In Vitro*	*In Vivo*
Increases Osteocalcin expression together with Nuclear related receptor-1 [8] Enhances Osteocalcin promoter activity interacting with Estrogen-related receptor alpha [9] Restores the inhibition of osteogenic differentiation and mitochondrial activity Sirtuin 3 knockdown-induced [10] Upregulates many key factors involved in osteoblast and osteocyte differentiation [11]	PGC1α deletion causes a reduction in cortical thickness and in *osteocalcin* and *collagen type* I α 1 levels [12] PGC1α/β deficiency results in cortical and trabecular parameter reduction [11] PGC1α absence induces marrow adipose tissue accumulation [13] PGC1α activation in leptin receptor-deficient diabetic mice increases osteoblastic gene expression and inhibits atrogene transcription [14]
